# Genome Sequences of *Comamonadaceae* Bacteria OS-1 and OS-4, Two Highly H_2_O_2_-Sensitive Strains Isolated from Pond Water

**DOI:** 10.1128/mra.01198-22

**Published:** 2023-02-13

**Authors:** Motoyuki Watanabe, Kensuke Igarashi, Souichiro Kato, Yoichi Kamagata, Wataru Kitagawa

**Affiliations:** a Graduate School of Agriculture, Hokkaido University, Sapporo, Hokkaido, Japan; b Graduate School of Global Food Resources, Hokkaido University, Sapporo, Hokkaido, Japan; c Bioproduction Research Institute, National Institute of Advanced Industrial Science and Technology, Sapporo, Hokkaido, Japan; d Bioproduction Research Institute, National Institute of Advanced Industrial Science and Technology, Tsukuba, Ibaraki, Japan; University of Arizona

## Abstract

The *Comamonadaceae* bacterial strains OS-1 and OS-4 were isolated from pond water and were found to be highly sensitive to hydrogen peroxide in the agar plates. Here, we report the nearly complete and complete genome sequences, respectively, of these two strains.

## ANNOUNCEMENT

The *Comamonadaceae* bacterial strains OS-1 and OS-4 were isolated as microbes that show unprecedented sensitivity to low micromolar levels of H_2_O_2_ in the agar plates (1). These strains were isolated on 12 July 2018 from the water of a pond belonging to Hokkaido University (Sapporo, Hokkaido, Japan) (43°04′27.5″N, 141°20′29.2″E). Their genome sequences are reported for further understanding of their high H_2_O_2_ sensitivities.

From the original frozen glycerol stocks, both strains were cultured on peptone-yeast-glucose (PYG) agar medium (2), and then single colonies were picked and grown for 5 days at 20°C in PYG liquid medium. The genomic DNA was extracted using the phenol-chloroform method (3), and the same DNA was used to prepare short- and long-read sequencing libraries. Short-read sequencing was performed using an iSeq 100 system with paired-end 150-bp reads, and the Nextera DNA Flex library preparation kit (Illumina) was used for sequencing library preparation. The reads were quality trimmed with fastp v0.12.4 (–q 15, –n 10, –t 1, –T 1, –l 20, –w 16, –g, with other parameters at the defaults) (4), which resulted in 4,535,428 paired-end reads for OS-1 and 4,052,006 paired-end reads for OS-4. Long-read sequencing was performed using a MinION Mk1B system, and the library was prepared with the SQK-LSK109 kit (Oxford Nanopore Technologies). The reads were base called using the high-accuracy mode of MinKNOW v4.1.22, followed by quality trimming using GNU Parallel v20181022 (5) and Nanofilt v2.7.1 (–quality 10, –length 500, –headcrop 75, with other parameters at the defaults) (6), and the quality was assessed with NanoStat v1.5.0 (6). As a result, 610,015 reads for OS-1 (read *N*_50_ value of 2,113 bases and total read length of 1,069,535,023 bases) and 393,661 reads for OS-4 (read *N*_50_ value of 3,489 bases and total read length of 1,130,220,753 bases) were obtained.

The hybrid assembly of iSeq and MinION reads was performed using SPAdes v3.14.1 (–k 21,33,41,55,77, –nanopore, –careful, with other parameters at the defaults) (7, 8). For OS-1, a single contig of >200 nucleotides (nt) was generated (length of 5,121,829 nt and coverage of 64×). This contig is a single chromosome; however, due to the presence of long tandem repeats on the ends of the contig, the circularity was not confirmed. For OS-4, 2 contigs of >200 nt were generated, i.e., a single chromosome (length of 3,763,098 nt and coverage of 68×) and a single plasmid (length of 318,875 nt and coverage of 109×). The ends of the contigs overlapped, and thus the circularities were confirmed by PCR amplification of the overlapping regions. The assembled sequences of both strains were annotated using Prokka v1.14.6 (9) with default parameters, and the chromosomal sequence of OS-4 was rotated manually to the start of the *dnaA* gene. The tandem repeats on the ends of the OS-1 chromosome contig were annotated as an rRNA operon, and at least two copies are present. The characteristics and accession numbers of the assembled and annotated sequences are presented in Table 1.

**TABLE 1:** Characteristics and DDBJ accession numbers for genome sequences

Parameter	Data for:		
	OS-1 chromosome	OS-4	
		Chromosome	Plasmid
Completeness	Nearly complete	Complete	Complete
Length (bp)	5,121,829	3,763,024	305,179
G+C content (%)	63.5	60.2	56.3
No. of genes	4,696	3,547	342
No. of protein-coding genes	4,627	3,501	342
No. of tRNA genes	56	42	0
No. of rRNA genes	12	3	0
No. of transfer-messenger RNA genes	1	1	0
DDBJ accession no.	AP026969	AP026970	AP026971

Both OS-1 and OS-4 strains contain putative genes of catalases and alkyl hydroperoxide reductases, which degrade H_2_O_2_. The results again demonstrated that microbes could be sensitive to low micromolar levels of H_2_O_2_ in the agar plate regardless of the possession of common H_2_O_2_-degrading genes, as we reported previously (10).

### Data availability.

The genome sequences have been deposited in DDBJ under the accession number AP026969 for OS-1 and the accession numbers AP026970 and AP026971 for OS-4. The raw sequence reads have been deposited in the DRA under the accession numbers DRX402456 (iSeq) and DRX402457 (MinION) for OS-1 and the accession numbers DRX402458 (iSeq) and DRX402459 (MinION) for OS-4.
